# Current understanding of sodium-glucose transporter 2 inhibitors in cardiovascular-kidney-metabolic syndrome

**DOI:** 10.3389/fphar.2026.1800868

**Published:** 2026-04-10

**Authors:** Yan Yang, Wen Zhou, Min Yang, Sydney C. W. Tang, Bi-Cheng Liu

**Affiliations:** 1 Department of Nephrology, Changzhou First People’s Hospital, Changzhou, China; 2 Division of Nephrology, Department of Medicine, The University of Hong Kong, Hong Kong SAR, China; 3 Institute of Nephrology, Zhongda Hospital, Southeast University School of Medicine, Nanjing, China

**Keywords:** cardiovascular disease, cardiovascular-kidney-metabolic syndrome, chronic kidney disease, metabolic syndrome, SGLT-2i

## Abstract

Cardiovascular-kidney-metabolic (CKM) syndrome is a recently defined clinical entity that encompasses cardiovascular disease (CVD), chronic kidney disease (CKD) and metabolic disorders. It has emerged as a growing public health concern that adversely affects the quality of life and imposes a substantial burden on human health. Sodium-glucose cotransporter 2 inhibitor (SGLT-2i) is a novel class of oral hypoglycemic agent with novel insulin-independent mechanism. In the last decade, published studies highlight its substantial effects on renal and cardiovascular outcomes. SGLT-2i is recommended for patients with stages 2–4 CKM syndrome, particularly those with CKD or diabetes to delay disease progression, and improve long-term clinical outcomes. This review comprehensively summarizes the current clinical evidence and elucidates the underlying mechanisms of SGLT-2i in CKM syndrome. Glycosuria and natriuresis, the primary effects of SGLT2 inhibition, play a pivotal role in improving glycemic control, reducing body weight, and lowering blood pressure. These initial effects trigger a cascade of downstream mechanisms: hemodynamic optimization via interstitial fluid reduction, enhanced cardiac efficiency through ketogenesis, and attenuation of inflammation and oxidative stress. Additional systemic benefits include increased fatty acid utilization, reduced hyperuricemia and stimulated erythropoiesis, thereby generating a network of interrelated therapeutic benefits in CKM syndrome. The pleiotropic effects of SGLT-2i position it as a highly promising therapeutic strategy for CKM syndrome. A deeper understanding of underlying mechanisms will better inform the application of SGLT-2i for this newly defined condition and guide optimal treatment strategies.

## 1 Introduction

Metabolic syndrome (MetS), cardiovascular disease (CVD), and chronic kidney disease (CKD) are common diseases that seriously threaten the life and health of human being (Guembe et al., 2020; Scurt et al., 2024). MetS is a critical risk factor for both CVD and CKD, and cardiorenal syndrome highlights the interaction between CVD and CKD. Accumulating evidence indicates that MetS, CVD, and CKD share overlapping pathophysiological pathways and mutually exacerbate one another, culminating in a progressive clinical trajectory. In response, the American Heart Association (AHA) formally introduced the conceptual framework of cardiovascular-kidney-metabolic (CKM) syndrome, defining it as a systemic disease characterized by the pathophysiological interactions among metabolic risk factors, CKD, and the cardiovascular system. Central to this framework is the recognition of a vicious cycle driven by shared mechanisms, including insulin resistance and metabolic dysfunction, chronic low-grade inflammation, maladaptive neurohormonal activation, as well as endothelial dysfunction and accelerated atherosclerosis (Ndumele et al., 2023a). The population of CKM syndrome contains individuals at risk for CVD as well as patients with CVD and CKD (Ndumele et al., 2023a; Ndumele et al., 2023b). Epidemiological data reveal a high population burden, with nearly 90% of U.S. adults meeting CKM criteria and 14.6% presenting with advanced stages (stage 3 or 4) (Aggarwal et al., 2024). Patients with concurrent DM, CKD, and CVD have a poor prognosis (Kadowaki et al., 2022), as these diseases interact with each other to create a vicious cycle. Therefore, it is the key to implement holistic interventions targeting multiple diseases simultaneously in CKM syndrome.

Sodium-glucose cotransporter 2 (SGLT-2), a high-capacity, low-affinity glucose transporter primarily expressed in the S1/S2 segments of renal proximal tubular epithelial cells, mediates approximately 90% of renal glucose reabsorption (Ghezzi et al., 2018). SGLT-2 inhibitor (SGLT-2i) is a new class of oral hypoglycemic agents that derived from the discovery of phlorizin. It selectively inhibits SGLT-2 to reduce glucose reabsorption at proximal renal tubules, promoting urinary glucose excretion and consequently lowering blood glucose levels in an insulin-independent manner (Ghezzi et al., 2018). More interestingly, it has been widely demonstrated to bear additional therapeutic benefits such as weight loss, uric acid lowering and blood pressure reduction. Several cardiovascular outcome trails (CVOTs) have indicated that SGLT-2i reduce the composite cardiovascular and renal end points in patients with T2DM (Zinman et al., 2015; Neal et al., 2017; Wiviott et al., 2019). Notably, randomized controlled trials (RCTs) have confirmed significant efficacy of SGLT-2i in heart failure (HF) and CKD, regardless of the presence of T2DM (Packer et al., 2020; Heerspink et al., 2020a; Solomon et al., 2022). Based on these pleiotropic effects, SGLT-2i is proposed to have great potential for the treatment of CKM syndrome. Nonetheless, the exact mechanisms underlying the major organ protective effects of SGLT-2i on CKM syndrome are not fully elucidated. Here we provided a comprehensive review for the current understanding of SGLT-2i on this newly defined critical clinical issue.

## 2 Clinical application of SGLT-2i

### 2.1 Application of SGLT-2i in metabolic syndrome

MetS, characterized by a constellation of metabolic disorders including obesity, hyperglycemia, dyslipidemia, hypertension, and hyperuricemia, is primarily driven by insulin resistance and hyperinsulinemia secondary to central obesity. Substantial evidence from clinical trials supports the broad therapeutic effects of SGLT-2i on these components. Key CVOTs consistently reported that empagliflozin, canagliflozin, and dapagliflozin significantly lowered HbA1c, body weight, systolic and diastolic blood pressure in patients with T2DM (Zinman et al., 2015; Neal et al., 2017; Wiviott et al., 2019). A meta-analysis of 8 RCTs further confirmed significant weight reduction with SGLT-2i in non-diabetic obese individuals (Wong et al., 2021). Moreover, SGLT-2i effectively reduced serum uric acid levels in both diabetic and non-diabetic populations, indicating potential utility in hyperuricemia management (Yip et al., 2022; Yokose et al., 2024). Regarding lipid metabolism, SGLT-2i generally increased high-density lipoprotein cholesterol (HDL-C) while lowering total cholesterol and triglycerides (Zinman et al., 2015; Hayashi et al., 2017). However, their effect on low-density lipoprotein cholesterol (LDL-C) remains nuanced, with evidence suggesting a shift toward less atherogenic LDL subfractions (Hayashi et al., 2017; Bechmann et al., 2024; Ding and Rexrode, 2020). Emerging data also indicate that empagliflozin decreased alanine transaminase reduced hepatic fat content in patients with T2DM and metabolic dysfunction-associated steatotic liver disease (MASLD) (Amin et al., 2025). Collectively, these findings underscore the multifaceted benefits of SGLT-2i in ameliorating the key metabolic abnormalities that define MetS.

### 2.2 Application of SGLT-2i in chronic kidney disease

The EMPA-REG OUTCOME, CANVAS, and DECLARE-TIMI 58 CVOTs have confirmed the favorable renal protective effects of SGLT-2i (Zinman et al., 2015; Neal et al., 2017; Wiviott et al., 2019). Subsequent trials, including CREDENCE and DELIGHT, extended these benefits to patients with T2DM and established impaired renal function, and found that the glucose-lowering effect was diminished in these patients (Perkovic et al., 2019; Pollock et al., 2019). The landmark DAPA-CKD trial demonstrated that dapagliflozin significantly reduced the composite renal risk by 39% (HR = 0.61, 95%CI = 0.51–0.72) in patients with CKD (Heerspink et al., 2020a). Prespecified analysis showed no heterogeneity in the impact of dapagliflozin on the primary outcome across different causes of CKD and diabetes status (Wheeler et al., 2021). It also notably reduced urinary albumin-to-creatinine ratio (UACR) by 29.3% (Jongs et al., 2021) and slowed the estimated glomerular filtration rate (eGFR) decline by 0.95 mL/min/1.73 m^2^/year (Heerspink et al., 2021). Similarly, the EMPA-KIDNEY trial confirmed a 29% reduction in kidney disease progression risk with empagliflozin (The EMPA-KIDNEY Collaborative Group et al., 2023), with benefits persisting into the post-trial period (EMPA-KIDNEY Collaborative Group et al., 2025). These findings are corroborated by real-world evidence, such as the CVD-REAL 3 study (Heerspink et al., 2020b).

Notably, empagliflozin specifically reduced fluid overload without altering adipose tissue mass in CKD patients, as shown in a bioimpedance substudy of EMPA-KIDNEY (Mayne et al., 2024). Consequently, current KDIGO guidelines strongly recommend SGLT-2i for patients with T2DM and CKD (eGFR ≥20 mL/min/1.73 m^2^) to improve cardiorenal outcomes (1A) (Rossing et al., 2022). While a systematic review supports their efficacy across various baseline CKM conditions (Siddiqi et al., 2025), key questions remain regarding the optimal timing of initiation and their role in advanced kidney disease (e.g., dialysis, transplantation, or eGFR <20 mL/min/1.73 m^2^), highlighting important avenues for future research.

### 2.3 Application of SGLT-2i in cardiovascular disease

SGLT-2i have demonstrated robust cardiovascular benefits across a wide spectrum of patients. Large COVTs in T2DM, including EMPA-REG OUTCOME (Zinman et al., 2015), CANVAS (Neal et al., 2017), DECLARE-TIMI 58 (Wiviott et al., 2019), and VERTIS CV (Cannon et al., 2020), consistently showed significant reductions in hospitalization for HF and, in some cases, cardiovascular death (Zinman et al., 2015), though effects on major adverse cardiovascular events (MACE) varied. The different included populations and different selectivity of gliflozins for SGLT-2 may lead to the neutral MACE results, but a clear explanation is still unclear. Subsequent trials extended these benefits to patients with established HF irrespective of diabetes status. DAPA-HF and EMPEROR-Reduced established the efficacy of SGLT-2i in reducing cardiovascular death and HF hospitalizations, and improving Kansas City Cardiomyopathy Questionnaire (KCCQ) scores in patients with HF with reduced ejection fraction (HFrEF) (Packer et al., 2020; McMurray et al., 2019; Curtain et al., 2021). Patients with HF with preserved ejection fraction (HFpEF) or mildly reduced ejection fraction (HFmrEF) showed a poor prognosis, with a 5-year mortality rate of approximately 40% (Jones et al., 2019). Current therapeutic options for HFpEF and HFmrEF are limited. Critically, the EMPEROR-Preserved and DELIVER trials demonstrated similar benefits in patients with HFpEF or HFmrEF (Solomon et al., 2022; Anker et al., 2021; Butler et al., 2022), establishing SGLT-2i as the first drug class effective across the full left ventricular ejection fraction (LVEF) spectrum. Further evidence from SOLOIST-WHF (Bhatt et al., 2021a) and SCORED (Bhatt et al., 2021b) supported their utility in high-risk settings, including recent HF hospitalization and comorbid CKD. Furthermore, a meta-analysis demonstrated that SGLT-2i prolonged survival, reduced hospitalizations or urgent visits for HF, and improved overall health status, with consistent benefit trends across different LVEF groups (Vaduganathan et al., 2022). These results indicate the efficacy of SGLT-2i in patients with HF is independent of LVEF.

Consequently, major guidelines (AHA/ACC/HFSA and ESC) now recommend SGLT-2i as foundational therapy for HF across phenotypes and for patients with T2DM and cardiorenal disease (Heidenreich et al., 2022; Marx et al., 2023). The major RCTs evaluating the effects of SGLT-2i on renal and cardiovascular outcomes are summarized in Supplementary Tables 1, 2. Meta-analyses and real-world data confirm these benefits extend to a broad population, including those without diabetes, across kidney function levels, and in conditions such as acute myocardial infarction (AMI) (Ceriello et al., 2023; Butler et al., 2024; Marfella et al., 2023; Marfella et al., 2024; Liu et al., 2025; Patel et al., 2024). Collectively, the evidence positions SGLT-2i as a pivotal therapeutic strategy within CKM syndrome management, particularly for patients with coexisting CKD and HF. However, the precise mechanistic pathways underlying their pleiotropic benefits warrant further investigation.

## 3 The mechanisms of SGLT-2i on metabolic risk factors

### 3.1 Anti-hyperglycemic effect

In normal adults, approximately 180 g glucose is filtered by the glomeruli daily and completely reabsorbed by the renal tubules, accounting for about one-third of the body’s energy expenditure. SGLT-2i acts by competitively binding to SGLT-2 proteins, thereby inhibiting glucose reabsorption in the proximal tubules (Ghezzi et al., 2018). SGLT-2i reduce the renal threshold for glucose excretion that induce a sustained urinary glucose loss of 40–80 g per day, leading to a reduction of HbA1c by 0.5%–0.7% in patients with T2DM (Vallon and Thomson, 2017). The consequent increase in urinary glucose content improves glycemic control and increases the genitourinary infection risk. Importantly, the hypoglycemic mechanism of SGLT-2i does not rely on the regulation of insulin resistance in the body or insulin secretion by pancreatic β-cells (Cowie and Fisher, 2020). SGLT-2i transfers glucose reabsorption downstream, where SGLT1 compensates and reduces the risk of hypoglycemia (Vallon and Thomson, 2017).

### 3.2 Inhibiting obesity

SGLT-2i increases urinary glucose excretion, leading to a daily energy loss of approximately 200–250 kcal in the urine, initially lose weight through an osmotic diuretic effect that reduces body water (Cowie and Fisher, 2020). Subsequently, SGLT-2i induces a shift in energy substrate utilization from carbohydrates toward lipids and ketones, which contributes to a reduction in body fat, including both visceral and subcutaneous adipose tissue (Vallon and Thomson, 2017). The body weight reduction was observed in the first week of SGLT-2i therapy, plateaued and maintained after 6 months due to a gradual compensatory increase in caloric intake (Cowie and Fisher, 2020). Studies using bioimpedance spectroscopy confirmed that medium-term weight loss under SGLT-2i was primarily attributable to reductions in adipose tissue mass, with preservation of muscle mass and bone mineral content, and a transient loss of extracellular fluid (Schork et al., 2019).

The effect of SGLT2i on adipose tissue metabolism has attracted increasing research attention. Canagliflozin has been proven to upregulate genes related to mitochondrial biogenesis and fatty acid (FA) oxidation (Wei et al., 2020). In murine models, SGLT-2i increased hepatic and plasma levels of fibroblast growth factor 21 (FGF-21), a key regulator of lipid oxidation, white adipose tissue (WAT) browning, and adiposity reduction (Osataphan et al., 2019; Xu et al., 2017). Additionally, in hereditary hypertriglyceridemic rats, empagliflozin enhanced insulin sensitivity in WAT, reduce adipose tissue mass, and suppress weight gain by upregulating gluconeogenesis-related genes while downregulating lipogenic genes in the hepatic, renal, and adipose tissue (Trnovska et al., 2021). Together, these findings demonstrate that SGLT-2i plays a critical role in mitigating adipose tissue dysfunction and obesity.

### 3.3 Lowering blood pressure

SGLT-2i lowers both systolic and diastolic blood pressure without increasing heart rate. The early antihypertensive mechanism is primarily due to osmotic natriuresis and diuresis, which leads to a sustained decrease in plasma volume. Sodium-hydrogen exchanger 3 (NHE3) is another major contributor in Na^+^ reabsorption and works synergistically with SGLT-2 which can regulate its activity. Castro et al. found that SGLT-2i reduced NHE3 activity in the renal proximal tubule of spontaneously hypertensive rats without significantly affecting other apical tubular transporters and channels (Castro et al., 2024). However, the mechanism through which SGLT-2i reduces NHE3 activity has not been determined. Long-term mechanisms for blood pressure reduction include modulation of the renin-angiotensin-aldosterone system (RAAS) and the sympathetic nervous system (SNS) (Puglisi et al., 2021; Herat et al., 2020), reduction in visceral and subcutaneous adipose tissue, and lower uric acid levels. Nevertheless, in a nondiabetic model of salt-sensitive hypertension, dapagliflozin blunted hypertension development by glucosuria and natriuresis without changes of hormone levels in the RAAS or the expression and activity of other Na^+^ channels and transporters (Kravtsova et al., 2022). Besides, SGLT-2i ameliorated arterial pressure lability through improvement of baroreflex sensitivity and weakened sympathetic nerve activity in streptozotocin-induced diabetic rats (Yoshikawa et al., 2017). Bosch et al. identified change in systolic 24-h ambulatory blood pressure triggering the improvement of arterial stiffness in patients with T2DM (Bosch et al., 2019). SGLT-2i improves endothelial function and arterial stiffness through inhibiting advanced glycation end products (AGEs) expression, increasing nitric oxide (NO) synthesis and bioavailability, further reducing blood pressure (Cowie and Fisher, 2020).

### 3.4 Reducing uric acid

Many clinical studies suggested that reduction in plasma uric acid level could make a minor impact on cardiovascular benefits (Zinman et al., 2015; Yokose et al., 2024). Meta-analysis indicated that serum urate reduction effect of SGLT-2i were not significant in T2DM patients with CKD (Yip et al., 2022). SGLT-2i increases glucose concentration in the proximal tubules, where they compete with uric acid for glucose transporter 9 (GLUT9), leading to decreased reabsorption and increased excretion of uric acid, thereby reducing blood uric acid levels (Packer, 2020). This partly explains the weak serum urate reduction effect in CKD patients. Because of reduced uric acid filtration secondary to lower eGFR, SGLT-2i exerts weakened effects on decreased uric acid reabsorption. SGLT-2i also inhibits the enzyme xanthine oxidase, which catalyzes the formation of uric acid (Packer, 2020). In addition, Billing et al. suggested there was a physical association between SGLT2 and urate transporter 1 (URAT1) in the renal proximal tubule (Billing et al., 2024). The hypoglycemic effect of SGLT-2i influences the dose of endogenous insulin, reducing uric acid reabsorption by decreasing the activity of URAT1, further lowering blood uric acid (Novikov et al., 2019).

### 3.5 Improving lipid metabolism

SGLT-2i modulates lipid metabolism by regulating key molecules involved in lipid synthesis, transport, and FA oxidation. In high-fat diet (HFD)-induced obese mice, canagliflozin was shown to upregulate peroxisome proliferator-activated receptor-α (PPAR-α) while suppressing peroxisome proliferator-activated receptor-γ (PPAR-γ) and diacylglycerol O-acyltransferase 2 (DGAT2), the latter being integral to triglyceride synthesis and hepatic lipid accumulation (Ji et al., 2017). PPAR-α activation under energy-deficient conditions enhances FA uptake, binding, and β-oxidation, whereas PPAR-γ regulates FA storage and adipocyte differentiation (Wei et al., 2020). PPAR-γ upregulation also increases cluster of differentiation 36 (CD36) enzyme expression, promoting FA storage in adipocytes and adiponectin release, which further improves insulin sensitivity (Aragón-Herrera et al., 2019).

Empagliflozin treatment in rats reduced CD36 gene and protein expressions in atrial tissue, suggesting a role in ameliorating impaired cardiac autophagy—a mechanism potentially beneficial in cardiometabolic diseases (Aragón-Herrera et al., 2019). Preclinical studies further indicated that SGLT-2i induced a fasting-like metabolic state via FGF-21–dependent pathways, stimulating lipolysis, hepatic FA oxidation, and ketogenesis (Osataphan et al., 2019). Transcriptomic-metabolomic analysis revealed that canagliflozin activated adenosine 5′-mono-phosphate (AMP)-activated protein kinase (AMPK) and inhibited mammalian target of rapamycin (mTOR), modulating cellular nutrient sensing (Osataphan et al., 2019). Consistent with this, empagliflozin elevated AMPK and phosphorylated acetyl-CoA carboxylase (ACC) in skeletal muscle, promoting FA oxidation and ketogenesis while inhibiting cholesterol synthesis (Xu et al., 2017). Enhanced lipid oxidation may reduce toxic intracellular lipid metabolites, thereby alleviating lipotoxicity and improving insulin sensitivity and β-cell function in muscle (Cowie and Fisher, 2020; Xu et al., 2017). Future studies should clarify whether preferential myocardial ketone oxidation confers an energetic advantage in cardiometabolic disease.

### 3.6 Amelioration of liver steatosis

The pathophysiological overlap between MASLD and CVD has recently attracted widespread attention within the framework of cardiovascular-liver-metabolic health (CLMH) (Chew et al., 2025). Accumulating preclinical evidence suggested that SGLT-2i exhibited potential in mitigating MASLD progression. In rodent models, dapagliflozin was shown to preserve pancreatic β-cell mass and prevent hepatic steatosis (Omori et al., 2019), while the novel SGLT-2i NGI001 attenuated adipocyte hypertrophy, regulated adipokine secretion, and reduced hepatic lipid accumulation and inflammation (Chiang et al., 2020). These effects were associated with enhanced AMPK phosphorylation and subsequent phosphorylation of ACC-CoA, promoting FA oxidation (Chiang et al., 2020; Li et al., 2021).

Mechanistic studies indicated that SGLT-2i suppressed hepatocellular glucose uptake and reduce O-GlcNAcylation by inducing autophagy through AMPK-TFEB activation (Chun et al., 2023). Additionally, SGLT-2i modulated the AMPK-mTOR pathway to upregulate autophagy markers and inhibited lipogenesis and inflammation, thereby ameliorating liver injury (Li et al., 2021; Ma et al., 2022). Some evidence also points to antifibrotic effects via inhibition of the transforming growth factor-β (TGF-β) pathway through downregulation of miR-34a-5p and targeting of GREM2 (Shen et al., 2023). Immunomodulatory benefits have been observed, with empagliflozin reducing hepatic CD8^+^ T cell infiltration and suppressing T cell activation, potentially through enhanced ketogenesis (Liu et al., 2024). However, findings are not entirely consistent, as one study reported only mild improvement in hepatic fibrosis without significant changes in histology or gene expression (Makri et al., 2025). Collectively, these data indicate that SGLT-2i holds therapeutic promise for attenuating MASLD development, though further clinical validation is warranted.

## 4 Mechanisms of SGLT-2i for renal protection

### 4.1 Restoration of tubuloglomerular feedback

**FIGURE 1:**
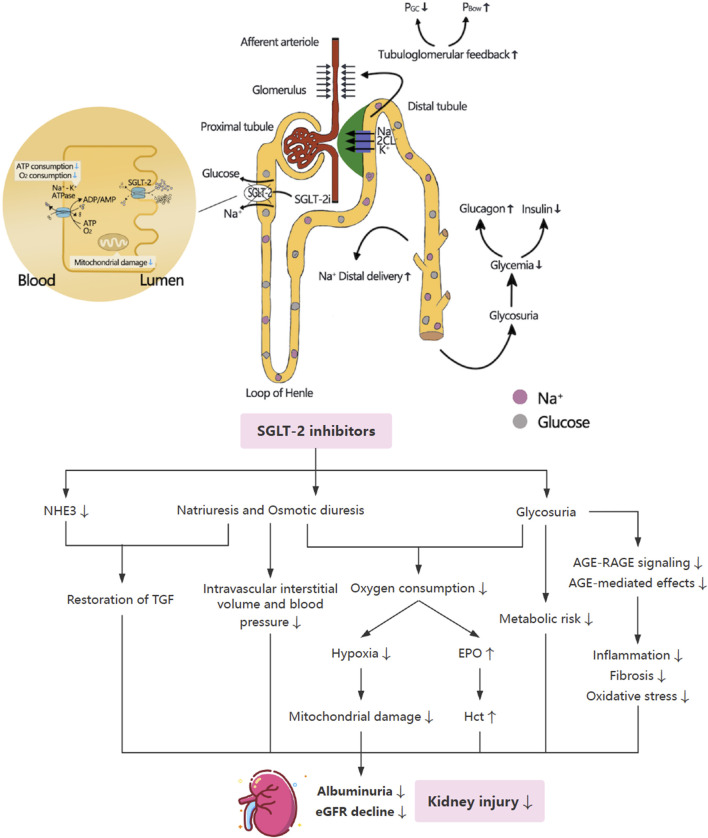
Potentially favorable effects of SGLT-2i on renal protection. SGLT-2i inhibits the resorption of Na^+^ and glucose, enhance Na^+^ delivery to macula densa and hence activate TGF, which increases P_Bow_ and decrease P_GC_ owing to afferent arteriole constriction, thereby improving glomerular hyperfiltration and reducing intraglomerular hypertension. SGLT-2i inhibits the activation of Na^+^-K^+^-ATPase via decreased Na^+^ and glucose resorption, thus leading to reduction in ATP and oxygen consumption, and alleviating hypoxia and mitochondrial damage. NHE, sodium-hydrogen exchanger; TGF, tubuloglomerular feedback; P_Bow_, pressure in Bowman’s space; P_GC_, glomerular capillary pressure; eGFR, estimated glomerular filtration rate; EPO, erythropoietin; Hct, hematocrit; AGEs, advanced glycation end products; RAGE, receptor for advanced glycation end products.

SGLT-2i induces a biphasic change in glomerular filtration rate (GFR), featuring an initial decline followed by long-term preservation. By inhibiting glucose and sodium reabsorption in the proximal tubule, SGLT-2i increases NaCl, K^+^, and fluid delivery to the macula densa, thereby activating tubuloglomerular feedback (TGF) (Figure 1) (Vallon and Verma, 2021). TGF activation leads to afferent arteriolar constriction via adenosine A1 receptors and efferent arteriolar dilation via adenosine A2 receptors, collectively reducing intraglomerular pressure and glomerular hyperfiltration, while simultaneously increasing hydrostatic pressure in Bowman’s space (P_Bow_) (Vallon and Verma, 2021; Kidokoro et al., 2019). Additionally, SGLT-2i promotes Na^+^ excretion through phosphorylation-dependent inhibition of NHE3, contributing to systemic natriuresis and volume regulation (Onishi et al., 2020).

Micropuncture studies in diabetic rat models confirmed that SGLT-2i lowered glomerular capillary pressure (P_GC_) via TGF-dependent pathways (Thomson and Vallon, 2021). The long-term renal protective effect is attributed to reduced mechanical stress on glomerular capillaries, decreased filtration of nephrotoxic macromolecules (e.g., albumin, growth factors, and advanced glycation end products), and attenuation of tubular hypertrophy and inflammation (Vallon and Verma, 2021). Concurrently, compensatory tubular adaptation in remaining nephrons helps sustain glucose reabsorption capacity, thereby maintaining functional nephron mass over time (Vallon et al., 2020). Taken together, these mechanisms underlie the ability of SGLT-2i to slow progressive renal function decline.

### 4.2 Improving hypoxia and stimulating EPO production

SGLT-2i inhibits the activation of Na^+^-K^+^-ATPase via decreased Na^+^ and glucose resorption, thus leading to reduction in ATP and oxygen consumption. The shift of glucose and Na^+^ reabsorption to the downstream S3 segment and the thick ascending limb of the loop of Henle can increase oxygen demand (Q_O2_) and lower medullary oxygen tension (P_O2_) outside the medulla (Vallon and Verma, 2021). Moreover, lower P_O2_ in the outer medulla activates hypoxia-inducible factors (HIF) and enhances the release of erythropoietin (EPO) (Vallon and Verma, 2021). Increased EPO, reticulocyte count, hemoglobin and hematocrit (Hct) levels were observed in T2DM and coronary arterial disease (CAD) patients in response to SGLT-2i (Mazer et al., 2020). This effect is associated with the inhibition of hepcidin and the modulation of iron-regulatory proteins, along with reducing the expression of HIF-1α and increasing the expression of its inhibitor, prolyl hydroxylase-2 (Ghanim et al., 2020). Furthermore, SGLT-2i reduces renal hypoxia by enhancing ketone bodies and potentially adipic acid metabolism, reducing blood glucose levels and glucose toxicity in the early proximal tubule, consequently improving tubular energetics as well as mitochondrial function and autophagy (Kidokoro et al., 2019; Tomita et al., 2020).

### 4.3 Other mechanisms

By shifting glucose, NaCl, and fluid reabsorption downstream, SGLT-2i balances transport workload along the nephron, which may help sustain tubular integrity over time. This is complemented by improved cortical oxygenation, reduced glucose toxicity, lower albumin filtration, and attenuated tubulointerstitial inflammation—collectively enhancing endothelial and tubular health and contributing to preserved GFR (Vallon and Verma, 2021). In the streptozotocin-induced diabetic rat model, dapagliflozin rescued the impairment of megalin-mediated albumin reabsorption in proximal tubules, thereby attenuating tubular albuminuria and mitigating subsequent tubulointerstitial injury (Farias et al., 2023). Hu et al. found that dapagliflozin attenuated tubulointerstitial fibrosis in hyperuricemic nephropathy (HN) patients and HN mice through activation of estrogen-related receptor α (ERRα)-organic anion transporter 1 (OAT1) axis (Hu et al., 2024). Similar results were also found in non-HN CKD mouse models and tubular epithelial cells treated with high-glucose or TGF-β (Hu et al., 2024). These data provided insights into SGLT-2i therapy of non-diabetic kidney diseases. However, other mechanisms of SGLT-2i alleviating renal fibrosis still need further studies.

Interestingly, it was noted that 2-week canagliflozin treatment altered microbiota composition and reduced plasma levels of p-cresyl sulfate and indoxyl sulfate in adenine-induced CKD mice model, even before renal function improvement (Mishima et al., 2018). Integrated multi-omics analyses in preclinical and clinical settings further support that SGLT-2i decreased microbiome-derived uremic toxin production (Billing et al., 2024). This suggests that modulation of the gut-kidney axis by SGLT-2i may contribute to renal protection. Further studies are needed to fully elucidate the interplay between SGLT-2i, intestinal microbiota, and renal outcomes.

## 5 Mechanisms of SGLT-2i for cardiovascular protection

### 5.1 Inducing a fasting response

SGLT-2i confers cardiorenal benefits cardiorenal benefits partly through the induction of a starvation-like metabolic state and subsequent stimulation of autophagy (Gao et al., 2022). By reducing cellular glucose availability, SGLT-2i activates nutrient deprivation signaling pathways—such as sirtuin (SIRT)/AMPK/peroxisome proliferator–activated receptor γ coactivator 1α (PGC-1α)—while simultaneously inhibiting nutrient surplus pathways, including protein kinase B (Akt)/mTOR (Mishima et al., 2018; Gao et al., 2022). This coordinated shift enhances autophagic flux, promoting cellular defense and survival mechanisms (Gao et al., 2022). Enhanced autophagy improves energy metabolism and fuel supply, reduces oxidative stress, cytotoxicity, inflammation, endoplasmic reticulum (ER) stress, and apoptosis, and contributes to the reduction of visceral and epicardial adipose tissue, thereby supporting the attenuation of HF progression (Packer, 2022).

### 5.2 Osmotic diuresis and urinary sodium excretion

**FIGURE 2:**
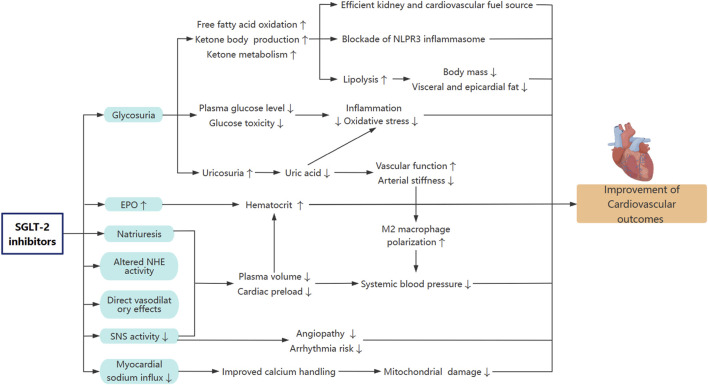
Potential mechanisms of SGLT-2i on cardiovascular protection. NHE, sodium-hydrogen exchanger; SNS, sympathetic nervous system; EPO, erythropoietin; NLRP3, nucleotide-binding domain-like receptor protein 3.

SGLT-2i exerts beneficial hemodynamic and volume-modulating effects in HF. Through mild osmotic diuresis and enhanced urinary sodium excretion, SGLT-2i improves volume status and reduce total body sodium. Unlike conventional diuretics, SGLT-2i preferentially clear fluid from the interstitial space while maintaining intravascular volume (Hallow et al., 2018), thereby minimizing neurohormonal activation and reducing cardiac preload (Figure 2). This mechanism is reflected by a sustained increase in Hct and improved natriuretic responsiveness, particularly in patients with volume overload (Mazer et al., 2020). In euvolemic HF, empagliflozin reduces skin sodium content—a marker of subclinical edema—with an initial natriuretic effect that attenuates over time, suggesting adaptive volume conservation (Kolwelter et al., 2023). Clinical data from the EMPEROR-Preserved trial indicate that empagliflozin facilitates diuretic down-titration (Butler et al., 2023). However, the EMPEROR-Reduced trial did not show superior efficacy in patients with overt fluid retention, implying that diuresis alone does not fully explain the cardiovascular benefits of SGLT-2i (Packer et al., 2021). These findings highlight the distinct volume-modulating properties of SGLT-2i, which contribute to—but are not the sole determinant of—their therapeutic effects in HF.

### 5.3 Improving myocardial energy metabolism

Caloric restriction, a hallmark of SGLT-2i, likely mediates their benefits, such as mitigating low-grade inflammation (Prattichizzo et al., 2021). SGLT-2i enhances myocardial energetics and efficiency through a shift in fuel metabolism, alongside anti-inflammatory, anti-fibrosis, and mitochondrial-improving actions, collectively ameliorating tissue hypoxia and cardiac function. Preclinical studies indicate that SGLT-2i-induced ketogenesis improves cardiac energy supply and power output (Santos-Gallego et al., 2019). While SGLT-2i may stimulate myocardial glucose uptake via glucose transporter 1 (Trum et al., 2020) or promote branched-chain amino acid metabolism (Kappel et al., 2017), recent evidence suggests that acute dapagliflozin treatment preferentially enhances cardiac ketone oxidation without substantially reducing FA or glucose oxidation (Goedeke et al., 2024), indicating that ketone bodies serve as a supplementary fuel source rather than displacing traditional substrates. Furthermore, SGLT-2i modulates cardiac lipid composition by reducing sphingolipids (sphingomyelin and ceramide) and glycerophospholipids, which are implicated in insulin resistance and cardiovascular injury. These alterations in myocardial lipid metabolism and accumulation are thought to play a key role in the pathogenesis of diabetic cardiomyopathy and HF, highlighting lipid remodeling as an important component of SGLT-2i-mediated cardio-protection.

### 5.4 Inhibiting the sympathetic nervous system

Sympathetic hyperactivity plays a key role in angiopathy, vasoconstriction, fatal arrhythmias, and the pathogenesis of HF, while SGLT-2i attenuates sympathetic nervous activity. Preclinical studies indicated that SGLT-2 inhibition reduced sympathetic markers such as tyrosine hydroxylase and norepinephrine, suggesting an attenuation of sympathetic outflow (Herat et al., 2020). However, clinical observations in patients with T2DM have not shown consistent changes in heart rate or muscle sympathetic nerve activity with empagliflozin, indicating that reflex-mediated sympathetic activation may not be a dominant clinical mechanism (Jordan et al., 2017). In contrast, the EMBODY trial demonstrated that empagliflozin significantly improved heart rate variability in patients with AMI and T2DM. This improvement, characterized by the changes in the standard deviation of all 5-min mean normal RR intervals and the low-frequency-to-high-frequency ratio, indicates a direct beneficial modulation of the delicate balance between cardiac sympathetic and parasympathetic nerve activity (Shimizu et al., 2020). Interestingly, individuals with a history of atrial fibrillation (AF) manifested a greater absolute benefit of SGLT-2i on cardiorenal events (Böhm et al., 2020). Supporting a direct antiarrhythmic potential, porcine models demonstrated that high-dose dapagliflozin terminated paroxysmal AF and promoted rhythm control in persistent AF (Paasche et al., 2024). Collectively, these findings point to a modulatory role of SGLT-2i on autonomic balance and cardiac electrophysiology, though the precise clinical significance and underlying mechanisms warrant further investigation.

### 5.5 Impact on myocardial cell ion homeostasis

While myocardial cells express SGLT1 rather than SGLT2, SGLT-2i appears to exert direct cardiac effects through off-target actions on cardiac ion transporters. A key mechanism involves inhibition of sodium-hydrogen exchanger 1 (NHE1), which is abundantly expressed in cardiomyocytes. By attenuating NHE1 activity, SGLT-2i reduces intracellular Na^+^ accumulation, thereby decreasing reverse-mode sodium-calcium exchanger activity and lowering cytosolic Ca^2+^ levels. This alleviates mitochondrial Ca^2+^ overload, improves mitochondrial antioxidant capacity, and mitigates myocardial injury (Baartscheer et al., 2017). Empagliflozin has been shown to reduce Ca^2+^/calmodulin-dependent kinase II (CaMKII) activity with consequent reduction in CaMKII-dependent sarcoplasmic reticulum Ca^2+^ leak and improvement of Ca^2+^ transients in isolated ventricular cardiomyocytes (Mustroph et al., 2018), contributing to the beneficial effects in HF. Of note, CaMKII inhibition can further suppress NHE1, creating a positive feedback loop that stabilizes intracellular ion homeostasis (Trum et al., 2020). Additionally, dapagliflozin directly inhibits peak sodium currents in atrial cardiomyocytes, reducing cellular excitability and exerting antiarrhythmic effects (Paasche et al., 2024). Computational deep learning and *in vivo* validations suggested that direct blockade of NHE1 by SGLT-2i restored the anti-apoptotic activity of X-linked inhibitor of apoptosis protein and baculoviral IAP repeat-containing protein 5, promoting cardiomyocyte survival independent of diabetic status (Iborra-Egea et al., 2019). These glucose-independent, ion-modulatory mechanisms collectively help restore the intracellular milieu, underscoring the pleiotropic cardioprotective potential of SGLT-2i. Further clinical validation is needed to fully establish these pathways in human HF and arrhythmia management.

### 5.6 Anti-inflammatory and anti-fibrotic effects

Chronic inflammation and fibrosis are central to CVD progression, and SGLT-2i exhibits multifaceted anti-inflammatory and anti-fibrotic properties. Preclinical studies demonstrate that SGLT-2i suppressed key inflammatory factors, including nuclear factor kappa-B (NF-κB), interleukin-6 (IL-6), tumor necrosis factor-α (TNF-α), monocyte chemoattractant protein-1 (MCP-1) (Yao et al., 2018; Ojima et al., 2015). Meanwhile, SGLT-2i reduced AGEs generation and suppressed AGE-RAGE signaling in diabetic rats and human cultured proximal tubular cells (Yao et al., 2018; Ojima et al., 2015). There was parallel reduction in oxidative stress, inflammatory and fibrotic reactions (Ojima et al., 2015). Furthermore, SGLT-2i attenuated nucleotide-binding domain-like receptor protein 3 (NLRP3) inflammasome activation in a Ca^+^ dependent manner, thereby reducing cardiac inflammatory cytokine release (Byrne et al., 2020). Interestingly, these anti-inflammatory effects may occur independently of changes in circulating ketone levels, suggesting a direct immunomodulatory role.

Cardiac fibrosis, driven largely by fibroblast activation and extracellular matrix dysregulation, is also mitigated by SGLT-2i. Empagliflozin significantly mitigated TGF-β1 induced fibroblast activation, reduced cell-mediated extracellular matrix remodeling responses, suppressed profibrotic markers such as p-Smad2, p-Smad3, and decreased cardiac connective tissue fraction (Kang et al., 2020; Li et al., 2019). These data suggested SGLT-2i ameliorated cardiac fibrosis by inhibiting the TGF-β1/Smad signaling pathway. HFD-fed mice and isolated cardiomyocytes experiments demonstrated empagliflozin modulated AMPK/mTOR signaling pathway mediated by Sestrin2 for reducing cardiac perivascular and interstitial fibrosis (Sun et al., 2020). However, some animal models of HFrEF and HFpEF showed functional improvement without significant changes in collagen content, indicating that fibrosis regression may not be essential for the early cardioprotective effects of SGLT-2i (Byrne et al., 2017; Connelly et al., 2019). Thus, while SGLT-2i clearly modulates inflammatory and fibrotic pathways, their precise role in cardiac fibrosis warrants further clarification.

### 5.7 Promoting cardiac remodeling

The EMPA-HEART CardioLink-6 trial found that empagliflozin significantly reduced left ventricular mass indexed to body surface area without concurrent improvement in systolic or diastolic function in patients with T2DM and CAD (Verma et al., 2019). These clinical data suggest that SGLT-2i have the potential to reverse cardiac remodeling, an effect that cannot be fully explained by the reduction in blood pressure alone. Mechanistically, SGLT-2i mitigates oxidative stress and mitochondrial dysfunction, key drivers of remodeling. Empagliflozin has been shown to enhance mitochondrial biogenesis and function via the PGC-1α/nuclear respiratory factor 1 (NRF-1)/mitochondrial transcription factor A pathway, improving atrial structure and electrical stability in diabetic models (Shao et al., 2019). In myocardial ischemia-reperfusion injury rat model, canagliflozin reduced myocardial infarct size and increased phosphorylation of endothelial NO synthase (eNOS) and AMPK, while phosphorylation of Akt was elevated only in infarcted hearts (Sayour et al., 2019). Additionally, dapagliflozin attenuated cardiac remodeling in obesity-related cardiomyopathy by inhibiting the MAPK/AP-1 pathway in an NHE1-dependent manner (Lin et al., 2022). Despite growing insights from both diabetic and non-diabetic models, direct comparative studies are needed to determine whether SGLT-2i operate through shared or distinct mechanistic pathways across different etiologies of cardiac remodeling.

### 5.8 Improving vascular function

Endothelial dysfunction, a central feature of CVD, is ameliorated by SGLT-2i. The meta-analysis showed SGLT-2i significantly improved the flow-mediated dilation levels in patients with T2DM, reflecting enhanced arterial endothelial function (Wei et al., 2022). Preclinical studies further elucidate underlying mechanisms: in HFpEF and obese rodent models, SGLT-2i restored endothelial nitric oxide synthase (eNOS) expression and activity, reduced vascular adhesion molecules and senescence markers, and improved endothelium-dependent relaxation (Cappetta et al., 2020; Park et al., 2020). Canagliflozin inhibited rat or human aortic vascular smooth muscle cells proliferation and migration by induction of heme oxygenase-1 (HO-1) expression and activity (Behnammanesh et al., 2020). Additionally, empagliflozin promoted macrophage polarization toward the anti-inflammatory M2 phenotype, which is critical event in atherosclerosis (Xu et al., 2017). Empagliflozin also improved cardiac microvascular perfusion through lessened vascular remodeling and endothelial function via improving eNOS phosphorylation and activation of AMPK pathway in diabetic mice and hyperglycemia-mediated cardiac microvascular endothelial cell (Zhou et al., 2018). Clinically, SGLT-2i improved arterial stiffness partially by reducing uric acid levels (Verma et al., 2020), and lowered Agatston calcification score in patients with T2DM (Wu et al., 2024). Vascular calcification animal models identified downregulation of thioredoxin domain-containing 5 (TXNDC5) and enhanced Runx2 degradation as potential mechanisms mitigating calcification (Wu et al., 2024). However, the effects of SGLT-2i on intimal calcification in endothelial cells and their cardiovascular benefits in non-diabetic populations remain to be fully established, warranting further investigation.

## 6 Adverse events of SGLT-2i therapy

Despite a generally favorable tolerance profile, SGLT-2i poses several clinically significant adverse effects (Supplementary Table 3). The risk of potentially serious complications may restrict their use in certain populations. Additionally, numerous minor side effects can compromise tolerability, which may subsequently diminish patient adherence and undermine the drug’s therapeutic efficacy. Consequently, safety and tolerability are critical considerations for prescribers.

### 6.1 Hypoglycemia

CVOTs indicated that hypoglycemia rarely occurred in SGLT-2i monotherapy (Packer et al., 2020; Solomon et al., 2022). However, combination with insulin or insulin secretagogues may increases hypoglycemia risk. Therefore, it is necessary to reduce dosage when combined with SGLT-2i.

### 6.2 Genitourinary infections

Genitourinary infections are the most frequent and prominent adverse effects of SGLT-2i due to increased urinary glucose concentration and potentially promoting pathogen growth (Vallon and Thomson, 2017). The network meta-analysis reported genital infection was largely specific to SGLT-2i [odds ratio (OR) = 3.30, 95%CI = 2.88 to 3.78] (Shi et al., 2023). Urinary tract infections often manifest as urethritis or cystitis, which are generally responsive to conventional antibiotic therapy. However, rare cases of severe infections such as sepsis, Fournier gangrene have been reported (Melnick et al., 2018).

### 6.3 Early eGFR decline

Since SGLT-2i acts on the nephron, impaired kidney disease can significantly modulate their activity. A well-established consequence is the attenuation of their glucose-lowering effect as GFR decreases (Vallon and Verma, 2021). Upon initiation of SGLT-2i, particularly in patients with advanced kidney disease or those receiving diuretics, a transient decline in eGFR>10% or rise in serum creatinine>30% may occur within 2–4 weeks in some individuals. Despite some post-marketing surveillance data from the FDA describing cases of acute kidney injury (AKI) following SGLT-2i initiation, subsequent large-scale analyses have shown no significant increase in AKI (Zinman et al., 2015; Neal et al., 2017; Wiviott et al., 2019).

### 6.4 Diabetic ketoacidosis

CVOTs have consistently reported adverse events of diabetic ketoacidosis (DKA), the overall incidence remained low and comparable to placebo in some RCTs (Zinman et al., 2015; Neal et al., 2017; Packer et al., 2020; Heerspink et al., 2020a; Solomon et al., 2022; Pollock et al., 2019; The EMPA-KIDNEY Collaborative Group et al., 2023; Cannon et al., 2020; McMurray et al., 2019; Anker et al., 2021; Bhatt et al., 2021a). The network meta-analysis revealed SGLT-2i increased DKA risk (OR = 2.07, 95%CI = 1.44–2.98) in patients with T2DM (Shi et al., 2023), though DKA requiring hospitalization remained rare. It is recommended to temporarily discontinue SGLT-2i in patients with severe infections, or hypovolemia, or undergoing surgery. If DKA occurs, SGLT-2i should be stopped, and management should include vigorous fluid replacement and intravenous insulin infusion. Once DKA is resolved, SGLT-2i therapy may be reintroduced (Chow et al., 2023).

### 6.5 Fracture, and amputation

CANVAS Program indicated an increased incidence of fracture (*P* = 0.02) and amputation (*P* < 0.001) in patients with T2DM and high cardiovascular risk receiving canagliflozin (Neal et al., 2017). However, empagliflozin and dapagliflozin have not demonstrated a significant increase in fracture and amputation risk. A real-world study showed a similar risk of fracture between SGLT-2i and glucagon-like peptide-1 receptor agonists (GLP-1RAs) (HR = 1.11, 95% CI = 0.93–1.33) in patients with T2DM, while SGLT-2i had a significantly higher risk of lower limb amputation (HR = 2.32, 95% CI = 1.37–3.91) (Rong et al., 2020). If patients develop obvious foot infections or skin ulcers, it is recommended to discontinue canagliflozin or switch to another SGLT-2i that does not increase risk of fractures or amputations (Neal et al., 2017).

### 6.6 Volume depletion and hypotension

The osmotic diuretic effect induced by SGLT-2i reduces extracellular volume by 5%–10% and lowers systolic blood pressure by 3–5 mmHg (Hallow et al., 2018). However, a meta-analysis showed that SGLT-2i did not increased risk of orthostatic hypotension [risk ratio (RR) = 1.17, 95% CI = 0.65 to 2.09] in patients with T2DM (Rong et al., 2020). CANVAS Program and DAPA-CKD trials indicated higher incidence of volume depletion in SGLT-2i compared to placebo (Neal et al., 2017; Heerspink et al., 2020a). A subgroup analysis of DAPA-HF study further revealed that volume depletion events during SGLT-2i occurred more frequently in patients concurrently receiving high-dose diuretics (McMurray et al., 2019).

### 6.7 Bladder cancer

Numerous clinical trials have investigated potential association between SGLT-2i and various cancer types. Higher incidence of newly diagnosed bladder cancer was reported in dapagliflozin (0.17% vs. 0.03%) (Hinnen, 2015). However, DECLARE-TIMI 58 trial reported lower incidence of bladder cancer in the dapagliflozin group than the placebo group (HR = 0.57, 95% CI = 0.35–93) (Wiviott et al., 2019). A comprehensive meta-analysis concluded that none of SGLT-2i was associated with a statistically significant increase in overall cancer risk (Pelletier et al., 2021). The long-term safety profile concerning physiological and morphological alterations in the urinary tract resulting from supersaturated glucose concentrations remains inadequately investigated.

## 7 Conclusion and future directions

**FIGURE 3:**
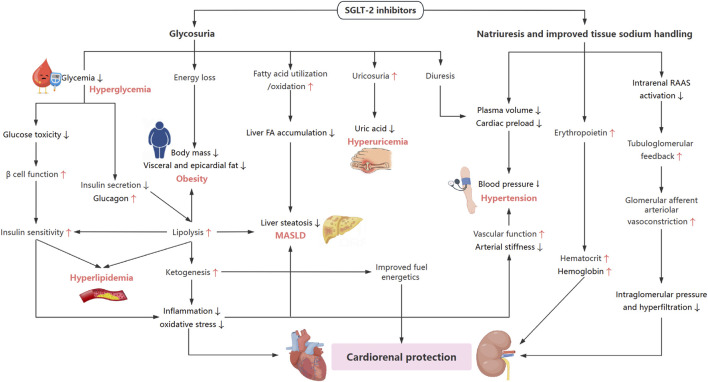
Schema of integrated beneficial effects of SGLT-2i on CKM syndrome. SGLT-2i ameliorates MASLD by inducing sustained glycosuria, subsequent increasing lipolysis and promoting FA oxidation, in conjunction with reduction in inflammation and oxidative stress. Potential common pathways of SGLT-2i on renal and cardiovascular benefits include reductions in blood pressure, reduced inflammation and oxidative stress, more efficient fuel with improved fuel energetics, and increased hematocrit. Restoration of TGF makes big contributions to renal protection. Further cardiac benefits may result from reduction in visceral and epicardial fat due to energy loss and increased lipolysis. FA, fatty acid; RAAS, renin-angiotensin-aldosterone system; MASLD, metabolic dysfunction-associated steatotic liver disease.

The development of SGLT-2i represents a significant advancement in the management of CKM syndrome, a major public health challenge that is closely associated with modern lifestyle changes. Current extensive clinical preclinical evidence supports the role of SGLT-2i in providing cardiorenal protection and attenuating the metabolic disorders. As illustrated in Figure 3, the pleiotropic effect of SGLT-2i on CKM syndrome are fundamentally tied to modulation of metabolic risk factors–including lowering plasma glucose and uric acid, increasing natriuresis, and stimulating lipolysis and ketogenesis, subsequently giving rise to series unexpected beneficial effects on CKM syndrome.

Despite these promising multi-target effects of SGLT-2i, most current data originate from studies with diabetes. Their use in population with high CVD risk without T2DM, combination therapy with other cardioprotective or nephroprotective agents, and appropriate time for initiating SGLT-2i remain controversial. To better understand the effectiveness and tolerability of SGLT-2i on CKM syndrome, more strict designed studies focusing on CKM are urgently needed. Furthermore, as the epigenetics attract increasing attention, future studies are warranted to explore the epigenetic mechanisms of SGLT-2i on CKM. Such studies have the potential not only to improve outcomes for patients with MetS and its cardiorenal complications, but also to alleviate the substantial economic burden on healthcare system.
