# Supplementary material for: Current understanding of sodium-glucose transporter 2 inhibitors in cardiovascular-kidney-metabolic syndrome
Source: Front Pharmacol. 2026 Apr 10;17:1800868. doi: 10.3389/fphar.2026.1800868 (PMC13106506; doi:10.3389/fphar.2026.1800868)
Supplement: Supplementary file 1 [file Table1.docx]

**Supplementary Table legends**

Supplementary Table 1. Effects of SGLT-2i on renal outcomes in RCTs

| Study | Participants (N) | CKM stages | Follow–up Time (median) | Intervention | Study Outcomes | HR (95%CI) |  |
| --- | --- | --- | --- | --- | --- | --- | --- |
| EMPA-REG OUTCOME [8] | 7020 T2DM patients with high risk for cardiovascular events and eGFR≥30 mL/min/1.73 m^2^ | 2-4 | 3.1 years (2.6 years of treatment) | Empagliflozin | Composite of doubling of serum creatinine, renal replacement therapy, or death from renal disease | **0.54 (0.40–0.75)** |  |
|  |  |  |  |  | Doubling of the serum creatinine level | **0.56 (0.39–0.79)** |  |
|  |  |  |  |  | Initiation of renal replacement therapy | **0.45 (0.21–0.97)** |  |
|  |  |  |  |  | Progression to macroalbuminuria | **0.65 (0.54–0.72)** |  |
|  |  |  |  |  | Incident or worsening nephropathy | **0.61 (0.53–0.70)** |  |
|  |  |  |  |  | Incident albuminuria | 0.95 (0.87–1.04) |  |
| CANVAS-R Program [9] | 5812 T2DM patients with high risk for cardiovascular events and eGFR>30 mL/min/1.73 m^2^ | 2-3 | 126.1 weeks | Canagliflozin | Composite of 40% reduction in eGFR, renal replacement therapy, or death from renal causes | **0.60 (0.47–0.77)** |  |
|  |  |  |  |  | Lower progression of albuminuria | **0.73 (0.67–0.79)** |  |
| DECLARE-TIMI 58 [10] | 17160 T2DM patients with established atherosclerotic CVD or at risk for atherosclerotic CVD (only 7% with eGFR<60 mL/min/1.73 m^2^) | 2-4 | 4.2 years | Dapagliflozin | Composite of 40% reduction in eGFR, new ESRD, or death from renal or cardiovascular causes | **0.76 (0.67–0.87)** |  |
| DAPA-CKD [12] | 4304 diabetic (68%) or non- diabetic patients suffering from CKD (UACR 200–5000 mg/g and eGFR 25–75 mL/min/1.73 m^2^) | 2 | 2.4 years | Dapagliflozin | Composite of 50% sustained decline in eGFR, ESRD, or renal death | **0.56 (0.45–0.68)** | independent of diabetes |
| CREDENCE [21] | 4401 T2DM patients with albuminuria CKD (eGFR of 30 to <90 mL/min/1.73 m^2^) | 2 | 2.62 years | Canagliflozin | Composite of ESKD, doubling of serum creatinine, or death from renal or cardiovascular causes | **0.70 (0.59–0.82)** |  |
|  |  |  |  |  | Composite of ESKD, doubling of serum creatinine, or death from renal causes | **0.66 (0.53–0.81)** |  |
|  |  |  |  |  | ESKD | **0.68 (0.54–0.86)** |  |
|  |  |  |  |  | Doubling of serum creatinine level | **0.60 (0.48–0.76)** |  |
| DELIGHT [22] | 461 T2DM patients with albuminuria (UACR 30–3500 mg/g) and eGFR of 25–75 mL/min/1.73 m^2^ | 2 | 24 weeks | Dapagliflozin | Variation of albumin–to–creatinine ratio | –21.0% for dapagliflozin (p=0.011) |  |
|  |  |  |  |  |  | –38.0% for dapagliflozin + saxagliptin (p<0.0001) |  |
| EMPA-KIDNEY [26] | 6609 patients with eGFR 20–45 ml/min/1.73m^2^, or eGFR 45–90 ml/min/1.73m^2^ with UACR at least 200 mg/g | 2 | 2 years | Empagliflozin | Progression of kidney disease (defined as initiation of maintenance dialysis or kidney transplantation, a sustained decline in eGFR to <10mL/min/1.73m^2^, a sustained decline of eGFR≥40% from baseline, or death due to renal causes) | **0.71 (0.62–0.81)** | independent of diabetes |
| VERTIS CV [32] | 8246 T2DM patients with established CVD and eGFR ≥30 mL/min/1.73 m^2^ | 4 | 3 years | Ertugliflozin | Composite of death from renal causes, renal replacement therapy, or doubling of serum creatinine | 0.81 (0.63–1.04) |  |
| DAPA-HF [33] | 4744 patients with class II–IV HF and an EF ≤40% | 4 | 18.2 months | Dapagliflozin | Composite of 50% reduction in eGFR, ESKD or death from renal causes | 0.71 (0.44–1.16) |  |
| EMPEROR-Reduced [11] | 3730 diabetic or non-diabetic patients with class II–IV HF and an EF ≤40% | 4 | 16 months | Empagliflozin | Composite of 40% reduction in eGFR or ESKD | **0.50 (0.32–0.77)** |  |
| DELIVER [13] | 6263 patients with HF and a LVEF > 40% | 4 | 2.3 years | Dapagliflozin | Composite of 50% reduction in eGFR, ESKD or death from renal causes | 1.08 (0.79–1.49) |  |
| EMPEROR-Preserved [36] | 5988 diabetic or not diabetic patients with class II–IV HF and an EF >40% | 4 | 26.2 months | Empagliflozin | Composite of 40% reduction in eGFR or ESKD | 0.95 (0.73–1.24) |  |
| SCORED [39] | 10584 T2DM with CKD (eGFR 25–60 ml/min/1.73m^2^) and risks for CVD | 2-4 | 16 months | Sotagliflozin | First occurrence of a sustained decrease of ≥50% in the eGFR from baseline for ≥30 days, long-term dialysis, renal transplantation, or sustained eGFR of <15 ml/min/1.73 m2 for ≥30 days | 0.71 (0.46–1.08) |  |

T2DM, Type 2 diabetes mellitus; eGFR, estimated glomerular filtration rate; CKD, chronic kidney disease; UACR, urinary albumin-to-creatinine ratios; ESRD, end-stage renal disease; CVD, cardiovascular disease.

Supplementary Table 2. Effects of SGLT-2i on cardiovascular outcomes in RCTs

| Study | Participants (N) | CKM stages | Follow–up Time (median) | Intervention | Study Outcomes | HR (95%CI) |  |
| --- | --- | --- | --- | --- | --- | --- | --- |
| EMPA-REG OUTCOME [8] | 7020 T2DM patients with high risk for cardiovascular events and eGFR≥30mL/min/1.73 m^2^ | 2-4 | 3.1 years (2.6 years of treatment) | Empagliflozin | MACE: composite of death from cardiovascular causes, nonfatal myocardial infarction, or nonfatal stroke | **0.86 (0.74–0.99)** |  |
|  |  |  |  |  | Death from cardiovascular causes | **0.62 (0.49–0.77)** |  |
|  |  |  |  |  | Hospitalization for HF | **0.65 (0.50–0.85)** |  |
|  |  |  |  |  | Death from any cause | **0.68 (0.57–0.82)** |  |
|  |  |  |  |  | Nonfatal stroke | 1.24 (0.92–1.67) |  |
|  |  |  |  |  | Nonfatal myocardial infarction | 0.87 (0.70–1.09) |  |
| CANVAS Program [9] | 10142 participants with T2DM and high cardiovascular risk | 2-3 | 188.2 weeks | Canagliflozin | Composite of death from cardiovascular causes, nonfatal myocardial infarction, or nonfatal stroke | **0.86 (0.75–0.97)** |  |
|  |  |  |  |  | Death from cardiovascular causes | 0.87 (0.72–1.06) |  |
|  |  |  |  |  | Nonfatal myocardial infarction | 0.85 (0.69–1.05) |  |
|  |  |  |  |  | Nonfatal stroke | 0.90 (0.71–1.15) |  |
|  |  |  |  |  | Fatal or nonfatal myocardial infarction | 0.89 (0.73–1.09) |  |
|  |  |  |  |  | Fatal or nonfatal stroke | 0.87 (0.69–1.09) |  |
|  |  |  |  |  | Hospitalization for any cause | 0.94 (0.88–1.00) |  |
|  |  |  |  |  | Hospitalization for HF | **0.67 (0.52–0.87)** |  |
|  |  |  |  |  | Death from cardiovascular causes or hospitalization for HF | **0.78 (0.67–0.91)** |  |
|  |  |  |  |  | Death from any cause | 0.87 (0.74–1.01) |  |
| DECLARE-TIMI 58 [10] | 17160 T2DM patients with established atherosclerotic CVD or at risk for atherosclerotic CVD (only 7% with eGFR < 60 mL/min/1.73 m^2^) | 2-4 | 4.2 years | Dapagliflozin | Composite of cardiovascular death, myocardial infarction, or ischemic stroke | 0.93 (0.84–1.03) |  |
|  |  |  |  |  | Cardiovascular death or hospitalization for HF | **0.83 (0.73–0.95)** |  |
|  |  |  |  |  | Hospitalization for HF | **0.73 (0.61–0.88)** |  |
| EMPEROR-Reduced [11] | 3730 diabetic or non-diabetic patients with class II–IV HF and an EF ≤40% | 4 | 16 months | Empagliflozin | Cardiovascular death or hospitalization for worsening HF | **0.75 (0.65–0.86)** | independent of diabetes |
|  |  |  |  |  | Hospitalization for HF | **0.70 (0.58–0.85)** |  |
| DAPA-CKD [12] | 4304 diabetic (68%) or non-diabetic patients suffering from CKD (UACR 200–5000 mg/g and eGFR 25–75 mL/min/1.73 m^2^) | 2 | 2.4 years | Dapagliflozin | Composite of cardiovascular death and hospitalization for HF | **0.71 (0.55–0.92)** |  |
| DELIVER [13] | 6263 patients with HF and a LVEF > 40% | 4 | 2.3 years | Dapagliflozin | Composite of worsening HF (an unplanned hospitalization for HF or an urgent visit for HF) or cardiovascular death | **0.82 (0.73–0.92)** | independent of diabetes |
|  |  |  |  |  | Hospitalization for HF or an urgent visit for HF | **0.79 (0.69–0.91)** |  |
|  |  |  |  |  | Hospitalization for HF | **0.77 (0.67–0.89)** |  |
|  |  |  |  |  | Urgent visit for HF | 0.76 (0.55–1.07) |  |
|  |  |  |  |  | Cardiovascular death | 0.88 (0.74–1.05) |  |
|  |  |  |  |  | Total number of worsening HF events and cardiovascular deaths | **0.77 (0.67–0.89)** |  |
|  |  |  |  |  | Change in KCCQ total symptom score at month 8 | **1.11 (1.03–1.21)** |  |
|  |  |  |  |  | Death from any cause | 0.94 (0.83–1.07) |  |
| CREDENCE [21] | 4401 T2DM patients with albuminuria CKD (eGFR of 30 to <90 mL/min/1.73 m^2^) | 2 | 2.62 years | Canagliflozin | Composite of cardiovascular death, myocardial infarction, stroke, or hospitalization for HF or unstable angina | **0.74, (0.63-0.86)** |  |
|  |  |  |  |  | Composites of cardiovascular death or hospitalization for HF | **0.69, (0.57-0.83)** |  |
|  |  |  |  |  | Composite of cardiovascular death, myocardial infarction, or stroke | **0.80 (0.67–0.95)** |  |
|  |  |  |  |  | Hospitalization for HF | **0.61 (0.47–0.80)** |  |
|  |  |  |  |  | Cardiovascular death | 0.78 (0.61–1.00) |  |
| EMPA-KIDNEY [26] | 6609 patients with eGFR 20–45 ml/min/1.73m^2^, or eGFR 45–90 ml/min/1.73m^2^ with UACR at least 200 mg/g | 2 | 2 years | Empagliflozin | Composite of hospitalization for heart failure or death from cardiovascular causes | 0.84 (0.67–1.07) | independent of diabetes |
|  |  |  |  |  | Death from cardiovascular causes | 0.84 (0.60–1.19) |  |
| VERTIS CV [32] | 8246 T2DM patients with atherosclerotic CVD and eGFR ≥30 mL/min/1.73 m^2^ | 4 | 3 years | Ertugliflozin | Composite of cardiovascular death, myocardial infarction, or ischemic stroke | 0.97 (0.85–1.11) |  |
|  |  |  |  |  | Death from cardiovascular causes or hospitalization for HF | 0.88 (0.75–1.03) |  |
|  |  |  |  |  | Death from cardiovascular causes | 0.92 (0.77–1.11) |  |
|  |  |  |  |  | Hospitalization for HF | **0.70 (0.54–0.90)** |  |
| DAPA-HF [33] | 4744 patients with class II–IV HF and an EF ≤40% | 4 | 18.2 months | Dapagliflozin | Composite of worsening HF (hospitalization or urgent visit resulting in intravenous therapy for HF) or cardiovascular death | **0.74 (0.65–0.85)** | independent of diabetes |
|  |  |  |  |  | First worsening HF event | **0.70 (0.59–0.83)** |  |
|  |  |  |  |  | Cardiovascular death | **0.82 (0.69–0.98)** |  |
| EMPEROR-Preserved [36] | 5988 diabetic or not diabetic patients with class II–IV HF and an EF >40% | 4 | 26.2 months | Empagliflozin | Cardiovascular death or hospitalization for worsening HF | **0.79 (0.69–0.90)** | independent of diabetes |
|  |  |  |  |  | Hospitalization for HF | **0.71 (0.60–0.83)** |  |
| SOLOIST-WHF [38] | 1222 T2DM patients recently hospitalized for worsening HF | 4 | 9 months | Sotagliflozin | Cardiovascular deaths and hospitalization or urgent visits for HF | **0.67 (0.52–0.85)** |  |
|  |  |  |  |  | Cardiovascular death | 0.84 (0.58–1.22) |  |
|  |  |  |  |  | Death from any cause | 0.82 (0.59–1.14) |  |
| SCORED [39] | 10584 T2DM with CKD (eGFR 25–60 ml/min/1.73m^2^) and risks for CVD | 2-4 | 16 months | Sotagliflozin | Total number of deaths from cardiovascular causes, hospitalizations for HF, and urgent visits for HF | **0.74 (0.63–0.88)** |  |
|  |  |  |  |  | Total number of hospitalizations for HF and urgent visits for HF | **0.67 (0.55–0.82)** |  |
|  |  |  |  |  | Deaths from cardiovascular causes | 0.90 (0.73–1.12) |  |
|  |  |  |  |  | Total number of deaths from cardiovascular causes, hospitalizations for HF, nonfatal myocardial infarctions, and nonfatal strokes | **0.72 (0.63–0.83)** |  |
|  |  |  |  |  | Total number of deaths from cardiovascular causes, hospitalizations for HF, urgent visits for HF, and events of HF during hospitalization | **0.76 (0.65–0.89)** |  |
|  |  |  |  |  | Deaths from any cause | 0.99 (0.83–1.18) |  |
|  |  |  |  |  | Total number of deaths from cardiovascular causes, nonfatal myocardial infarctions, and nonfatal strokes | **0.77 (0.65–0.91)** |  |
| EMPACT-MI [45] | 6522 patients with acute myocardial infarction and at risk for HF | 4 | 17.9 months | Empagliflozin | A first hospitalization for HF or death from any cause | 0.90 (0.76–1.06) |  |
|  |  |  |  |  | A first hospitalization for HF | **0.77 (0.60–0.98)** |  |
|  |  |  |  |  | Death from any cause | 0.96 (0.78–1.19) |  |
|  |  |  |  |  | Total number of hospitalizations for HF or death from any cause | 0.87 (0.68–1.10) |  |
|  |  |  |  |  | Total number of nonelective cardiovascular hospitalizations or death from any cause | 0.92 (0.78–1.07) |  |
|  |  |  |  |  | Total number of hospitalizations for myocardial infarction or death from any cause | 1.06 (0.83–1.35) |  |
|  |  |  |  |  | Death from cardiovascular causes | 1.03 (0.81–1.31) |  |
|  |  |  |  |  | A first hospitalization for HF or death from cardiovascular causes | 0.90 (0.75–1.07) |  |
|  |  |  |  |  | Total number of hospitalizations for HF | **0.67 (0.51–0.89)** |  |

T2DM, Type 2 diabetes mellitus; eGFR, estimated glomerular filtration rate; HF, heart failure; CKD, chronic kidney disease; UACR, urinary albumin-to-creatinine ratios; EF, ejection fraction; LVEF, left ventricular ejection fraction; KCCQ, Kansas City Cardiomyopathy Questionnaire; CVD, cardiovascular disease; MACE: major adverse cardiovascular events.

Supplementary Table 3. Adverse event rates of SGLT-2i in RCTs.

| Study | EMPA-REG OUTCOME [8] | CANVAS Program [9] | DECLARE-TIMI 58 [10] | EMPEROR-Reduced [11] | DAPA-CKD [12] | DELIVER [13] | CREDENCE [21] | DELIGHT [22] | EMPA-KIDNEY [26] | VERTIS CV [32] | | DAPA-HF [33] | EMPEROR-Preserved [36] | SOLOIST-WHF [38] | SCORED [39] | EMPACT-MI [45] |
| --- | --- | --- | --- | --- | --- | --- | --- | --- | --- | --- | --- | --- | --- | --- | --- | --- |
| Intervention | Empagliflozin | Canagliflozin | Dapagliflozin | Empagliflozin | Dapagliflozin | Dapagliflozin | Canagliflozin | Dapagliflozin | Empagliflozin | Ertugliflozin 5mg | Ertugliflozin 15mg | Dapagliflozin | Empagliflozin | Sotagliflozin | Sotagliflozin | Empagliflozin |
| Serious adverse event | **38.2% vs. 42.4%,** P<0.001 | **104.3 vs. 120.0,** P=0.04 | **34.1% vs. 36.2%,** P<0.001 | 41.4% vs. 48.1% | **29.5% vs. 33.9%,** P=0.002 | 43.5% vs. 45.5% | **33.50% vs. 36.69%** | 8% vs. 11% | **35.2% vs. 37.7%** | 34.9% vs. 36.1% | 34.1% vs. 36.1% | 35.7% vs. 40.2% | 47.9% vs. 51.6% | 38.8% vs. 41.1% | 23.4% vs. 25.2% | 23.7% vs. 24.7% |
| Adverse event leading to discontinuation of trial regimen | **17.3% vs. 19.4%,** P<0.01 | 35.5 vs. 32.8, P=0.07 | **8.1% vs. 6.9%,** P=0.01 | — | 5.5% vs. 5.7%, P=0.79 | 5.8% vs. 5.8% | — | 3% vs. 5% | — | 7.5% vs. 6.8% | 7.3% vs. 6.8% | 4.7% vs. 4.9%, P=0.79 | 19.1% vs. 18.4% | 4.8% vs. 3.8% |  | 3.8% vs. 3.8% |
| Hypoglycemia | 27.8% vs. 27.9% | 50.0 vs. 46.4, P=0.20 | **0.7% vs. 1.0%,** P=0.02 | 1.4% vs. 1.5% | **0.7% vs. 1.3%,** P=0.04 | 0.2% vs. 0.2% | — | 37% vs. 32% | 2.3% vs. 2.3% | 28.0% vs. 28.8%, P=0.51 | 26.5% vs. 28.8%, P=0.06 | 0.2% vs. 0.2% | 2.4% vs. 2.6% | 4.3% vs. 2.8% | 1.0% vs. 1.0%, P=0.84 | 0.1% vs. 0.2% |
| Diabetic ketoacidosis | 0.1% vs. <0.1% | 0.6 vs. 0.3, P=0.14 | **0.1% vs. 0.1%,** P=0.02 | 0% vs. 0% | 0.1% vs. <0.1%, P=0.50 | 0.1% vs. 0% | **5.00% vs. 0.05%** | 1% vs. 0% | 0.2% vs. 0.0% | 0.3% vs. 0.1% | 0.4% vs. 0.1% | 0.1% vs. 0% | 0.1% vs. 0.2% | 0.3% vs. 0.7% | **0.6% vs. 0.3%,** P=0.02 | 0.1% vs. <0.1% |
| Amputation | — | **6.3 vs. 3.4,** P<0.001 | 1.4% vs. 1.3%, P=0.53 | 0.7% vs. 0.5% | 1.6% vs. 1.8%, P=0.73 | 0.6% vs. 0.8% | 3.18% vs. 2.87% | 1% vs. 0% | 0.8% vs. 0.6% | 2.0% vs. 1.6% | 2.1% vs. 1.6% | 0.5% vs. 0.5%, P=1.00 | 0.5% vs. 0.8% | 0.7% vs. 0.2% | 0.6% vs. 0.6%, P=0.89 | 0.3% vs. 0.2% |
| Fracture | 3.8% vs. 3.9% | **15.4 vs. 11.9,** P=0.02 | 5.3% vs. 5.1%, P=0.59 | 2.4% vs. 2.3% | 4.0% vs. 3.2%, P=0.22 | — | 3.05% vs. 3.10% | 1% vs. 1% | 4.0% vs. 3.7% | 3.6% vs. 3.6% | 3.7% vs. 3.6% | 2.1% vs. 2.1%, P=1.00 | 4.5% vs. 4.2% | 2.0% vs. 1.5% | 2.1% vs. 2.2%, P=0.68 | — |
| Volume depletion | 5.1% vs. 4.9% | **26.5 vs. 18.5,** P=0.009 | 2.5% vs. 2.4%, P=0.99 | 10.6% vs. 9.9% | **5.9% vs. 4.2%,** P=0.01 | 1.3% vs. 1.0% | — | 3% vs. 3% | 2.5% vs. 2.3% | 4.3% vs. 3.9% | 4.3% vs. 3.9% | 7.5% vs. 6.8%, P=0.40 | — | 9.4% vs. 8.8% | **5.3% vs. 4.0%,** P=0.003 | 1.1% vs. 1.2% |
| Hypotension | — | — | — | 9.4% vs. 8.7% | — | — | — | — | — | — | — | — | 10.4% vs. 8.0% | 6.0% vs. 4.6% | — | — |
| Symptomatic hypotension | — | — | — | 5.7% vs. 5.5% | — | — | — | — | — | — | — | — | 6.6% vs. 5.2% | — | — | — |
| Renal-related adverse event | — | 19.7 vs. 17.4, P=0.32 | — | — | 7.2% vs. 8.7%, P=0.07 | 2.3% vs. 2.5% | — | 3% vs. 4% | — | — | — | 6.5% vs. 7.2%, P=0.36 | — | 11.6% vs. 12.4% | — | — |
| Acute kidney injury | **1.0% vs. 1.6%,** P<0.05 | 3.0 vs. 4.1, P=0.33 | **1.5% vs. 2.0%,** P=0.002 | — | — | — | 3.91% vs. 4.46% | — | 3.2% vs. 4.1% | 1.7% vs. 2.2% | 1.9% vs. 2.2% | — | 12.1% vs. 12.8% | 4.1% vs. 4.4% | — | 0.8% vs. 1.3% |
| Genital infection | **6.4% vs. 1.8%,** P<0.001 |  | **0.9% vs. 0.1%,** P<0.001 | 1.7% vs. 0.6% | — | — | — | 3% vs. 0% | <0.1% vs. <0.1% |  |  | — | 2.2% vs. 0.7% | 0.8% vs. 0.2% | **2.4% vs. 0.9%,** P<0.001 | — |
| Genital infection in women |  | **68.8 vs. 17.5,** P<0.001 |  |  |  |  |  |  |  | **6.0% vs. 2.4%,** P<0.001 | **7.8% vs. 2.4%,** P<0.001 |  |  |  |  |  |
| Genital infection in men |  | **34.9 vs. 10.8,** P<0.001 |  |  |  |  |  |  |  | **4.4% vs. 1.2%,** P<0.001 | **5.1% vs. 1.2%,** P<0.001 |  |  |  |  |  |
| Urinary tract infection | 18.0% vs. 18.1% | 40.0 vs. 37.0, P=0.38 | 1.5% vs. 1.6%, P=0.54 | 4.9% vs. 4.5% | — | — | — | 3% vs. 3% | 1.6% vs. 1.6% | **12.2% vs. 10.2%,** P=0.02 | **12.0% vs. 10.2%,** P=0.03 | — | 9.9% vs. 8.1% | 4.8% vs. 5.1% | 11.5% vs. 11.5%, P=0.45 | — |
| Cancer | — |  | 5.6% vs. 5.7%, P=0.83 | — | — | — |  | — | — | — | — | — | — | 0.7% vs. 0.7% | 0.9% vs. 0.8%, P=0.60 | — |
| Bladder cancer |  | 1.0 vs. 1.1, P=0.74 | **0.3% vs. 0.5%,** P=0.02 |  |  |  | 0.45% vs. 0.41% |  |  |  |  |  |  |  |  |  |
| Breast cancer |  | 3.1 vs. 2.6, P=0.65 | 0.4% vs. 0.4%, P=0.92 |  |  |  | 1.05% vs. 0.41% |  |  |  |  |  |  |  |  |  |
| Renal-cell carcinoma |  | 0.6 vs. 0.2, P=0.17 | — |  |  |  | 0.05% vs. 0.23% |  |  |  |  |  |  |  |  |  |
| Thromboembolic event | 0.6% vs. 0.9% | 1.7 vs. 1.7, P=0.63 | — | — | — | — | — | — | — | — | — | — | — | 0% vs. 1.1% | 0.6% vs. 0.7%, P=0.46 | — |
| Hypersensitivity | — | 8.5 vs. 6.1, P=0.17 | 0.4% vs. 0.4%, P=0.57 | — | — | — | — | — | — | — | — | — | — | — | — | — |
| Hepatic event | — | 7.4 vs. 9.1, P=0.35 | 1.0% vs. 1.0%, P=0.60 | — | — | — | — | — | 0.4% vs. 0.4% | 0.2% vs. 0.3% | 0.2% vs. 0.3% | — | 3.8% vs. 5.2% | — | — | 0.2% vs. 0.1% |
| Pancreatitis | — |  | — | — | — | — |  | — | — |  |  | — | — | 0% vs. 0.5% | 0.2% vs. 0.5%, P=0.16 | — |
| Acute |  | 0.5 vs. 0.4, P=0.63 |  |  |  |  | 0.23% vs. 0.09% |  |  | 0.4% vs. 0.4% | 0.2% vs. 0.4% |  |  |  |  |  |
| Chronic |  | — |  |  |  |  | — |  |  | <0.1% vs. 0.2% | 0.1% vs. 0.2% |  |  |  |  |  |
| Hyperkalemia | — | 6.9 vs. 4.4, P=0.10 | — | — | — | — | 6.86% vs. 8.24% | — | 2.8% vs. 3.3% | — | — | — | — | 4.3% vs. 5.1% | — | — |
| Diarrhea | — | — | — | — | — | — | — | — | — | — | — | — | — | 6.1% vs. 3.4% | **8.5% vs. 6.0%,** P<0.001 | — |

CANVAS Program: event rate per 1000 patient-yr
